# Airway macrophages display decreased expression of receptors mediating and regulating scavenging in early cystic fibrosis lung disease

**DOI:** 10.3389/fimmu.2023.1202009

**Published:** 2023-06-29

**Authors:** Lisa J. M. Slimmen, Vincent D. Giacalone, Craig Schofield, Hamed Horati, Badies H. A. N. Manaï, Silvia C. Estevão, Luke W. Garratt, Limin Peng, Rabindra Tirouvanziam, Hettie M. Janssens, Wendy W. J. Unger

**Affiliations:** ^1^ Division of Respiratory Medicine and Allergology, Department of Pediatrics, Erasmus University Medical Centre, Rotterdam, Netherlands; ^2^ Laboratory of Pediatrics, Infection and Immunity Group, Department of Pediatrics, Erasmus University Medical Centre, Rotterdam, Netherlands; ^3^ Department of Pediatrics, Emory University School of Medicine and Children’s Healthcare of Atlanta, Atlanta, GA, United States; ^4^ Telethon Kids Institute, University of Western Australia, Perth, WA, Australia; ^5^ Department of Biostatistics and Bioinformatics, Rollins School of Public Health, Emory University, Atlanta, GA, United States

**Keywords:** cystic fibrosis, children, lung disease, inflammation, airway macrophages, neutrophils, scavenger receptor, resolution

## Abstract

**Background:**

Cystic fibrosis (CF) airway disease is characterized by chronic inflammation, featuring neutrophil influx to the lumen. Airway macrophages (AMs) can promote both inflammation and resolution, and are thus critical to maintaining and restoring homeostasis. CF AM functions, specifically scavenging activity and resolution of inflammation, have been shown to be impaired, yet underlying processes remain unknown. We hypothesized that impaired CF AM function results from an altered expression of receptors that mediate or regulate scavenging, and set out to investigate changes in expression of these markers during the early stages of CF lung disease.

**Methods:**

Bronchoalveolar lavage fluid (BALF) was collected from 50 children with CF aged 1, 3 or 5 years. BALF cells were analyzed using flow cytometry. Expression levels of surface markers on AMs were expressed as median fluorescence intensities (MFI) or percentage of AMs positive for these markers. The effect of age and neutrophilic inflammation, among other variables, on marker expression was assessed with a multivariate linear regression model.

**Results:**

AM expression of scavenger receptor CD163 decreased with age (p = 0.016) and was negatively correlated with BALF %neutrophils (r = -0.34, p = 0.016). AM expression of immune checkpoint molecule SIRPα also decreased with age (p = 0.0006), but did not correlate with BALF %neutrophils. Percentage of AMs expressing lipid scavenger CD36 was low overall (mean 20.1% ± 16.5) and did not correlate with other factors. Conversely, expression of immune checkpoint PD-1 was observed on the majority of AMs (mean PD-1^pos^ 72.9% ± 11.8), but it, too, was not affected by age or BALF %neutrophils. Compared to matched blood monocytes, AMs had a higher expression of CD16, CD91, and PD-1, and a lower expression of CD163, SIRPα and CD36.

**Conclusion:**

In BALF of preschool children with CF, higher age and/or increased neutrophilic inflammation coincided with decreased expression of scavenger receptors on AMs. Expression of scavenging receptors and regulators showed a distinctly different pattern in AMs compared to blood monocytes. These findings suggest AM capacity to counter inflammation and promote homeostasis reduces during initiation of CF airway disease and highlight new avenues of investigation into impaired CF AM function.

## 1 Introduction

Cystic fibrosis (CF) is an autosomal recessive disorder caused by mutations in the *CFTR* gene, resulting in impaired transport of chloride and bicarbonate across the cell membrane. While CF affects many organ systems, its most prominent features are progressive airway damage caused by chronic neutrophilic inflammation, and infection starting in infancy (1, 2). Not only is inflammation chronically present in CF airways, but it has also become increasingly evident that resolution of inflammatory events is impaired in CF (3, 4), making it difficult to revert to homeostasis.

Macrophages, the most abundant immune cell type in human lungs at steady state (5), play a key role in both the ramp-up and resolution phases of an inflammatory response, and as such are pivotal for maintaining lung immune homeostasis. While absolute numbers of macrophages are higher in CF than non-CF airways, their function is also affected in several ways (6). CF airway macrophages (AMs) display higher basal production of key pro-inflammatory cytokines such as TNFα and IL-6 (7), and the neutrophil chemoattractant IL-8 (8), and show a higher inflammatory response to microbial stimuli compared to non-CF AMs (7, 9). In contrast, phagocytic capacity of pathogens such as *Pseudomonas aeruginosa* appears lower in CF than non-CF AMs (10, 11). One proposed explanation is the altered expression of scavenger receptors (SRs).

SRs are involved in a broad range of pro-inflammatory and pro-resolution macrophage functions. The SR superfamily comprises a group of receptors that bind to self antigens to facilitate the uptake of apoptotic cells (efferocytosis) and acellular debris, or to non-self antigens to mediate pathogen uptake (12). Lower SR expression (MARCO, CD68 and CD206) on sputum macrophages has been reported in adult subjects with CF compared to age-matched non-CF controls (13). As these SRs mediate pathogen uptake, their low expression may account at least in part for the fact that phagocytosis of pathogens is significantly reduced in CF AMs (11). However, the impact of CF on SR-mediated efferocytosis and scavenging of acellular debris has not been thoroughly evaluated. The removal of these cellular byproducts plays a crucial role in the resolution phase of inflammation. In addition to SR activity, macrophage phagocytosis is also facilitated by opsonin receptors (OpsRs). While little information is available on OpsR expression in CF AMs, monocyte-derived macrophages (MDMs) from CF peripheral blood showed the same expression level of OpsRs CD16 and CD64 as MDMs from healthy donor blood (11).

Scavenging and phagocytic activities are not only determined by expression levels of SRs, but also by immune checkpoint molecules, including signal regulatory protein α (SIRPα) and its ligand CD47, and programmed cell death protein 1 (PD-1). SIRPα is a key regulator of efferocytosis, inhibiting scavenging activity when bound to its ligand and “don’t-eat-me signal” CD47 on non-apoptotic cells. The inhibitory receptor PD-1 is expressed on macrophages upon activation and is associated with macrophage functional downregulation (14, 15).

To date, studies of SR and OpsR expression on CF macrophages relate to either sputum macrophages from advanced CF disease, or MDMs which may not fully reflect AM phenotype in the airway lumen. Moreover, while the comparison between CF and non-CF macrophages provides valuable insights into CF-specific phenotypic changes, it does not account for the large variability in airway inflammation seen among CF patients, nor does it inform macrophage biology during the earliest stages of CF airway disease. Due to the continuous presence of high numbers of neutrophils in CF airways, macrophage function is not only affected during periods of acute pulmonary exacerbation, but also during periods of steady-state (4, 6). Yet, few studies differentiate between CF patients with regards to the intensity of airway inflammation at the time of sampling.

Overall, current evidence seems to point towards impaired phagocytic as well as impaired pro-resolution function in CF AMs. However, the mechanisms through which phagocytic and scavenging abilities are impaired, and how the expression of involved receptors is altered, remain largely unknown. We hypothesized that the previously described scavenging dysfunction of AMs in CF is related to expression changes in SRs (scavenging effectors) and immune checkpoint molecules (scavenging regulators). To test this hypothesis, we measured the expression of these markers on AMs from freshly collected bronchoalveolar lavage of children with CF aged 1-5 years. Our results provide insight into AM phenotype in the early stages of CF airway disease, and how AM ability to maintain immune homeostasis is affected by an increasingly neutrophil-dominated lung environment.

## 2 Materials and methods

### 2.1 Study design and subjects

Children diagnosed with CF by neonatal screening were included in a prospective, longitudinal early CF monitoring program in Rotterdam, the Netherlands (I-BALL: Inflammatory markers in Broncho-Alveolar Lavage to predict early CF Lung disease). As part of this clinical monitoring program, patients underwent a bronchoscopy with bronchoalveolar lavage (BAL) and chest CT at their annual check-up. Subjects were included at ages 1, 3 or 5. Samples used in this study were collected between January 2018 and January 2023. The study was approved by the local institutional review board. Written informed consent was obtained from parents or legal guardians. The I-BALL study is registered on clinicaltrial.gov (identifier: NCT02907788).

### 2.2 Chest CT

A free-breathing chest CT was performed at the same visit as the bronchoscopy, or at most 1 week apart. Chest CTs were analyzed without the observer having any clinical information about the subject, using the standardized Perth-Rotterdam Annotated Grid Morphometric Analysis for CF (PRAGMA-CF) scoring (16). Percentage of diseased lung volume (PRAGMA %Dis) was calculated as the sum of percentages lung volume with bronchiectasis, mucus plugging and airway wall thickening.

### 2.3 Bronchoalveolar lavage fluid collection and processing for flow cytometry

Bronchoscopy was performed under general anesthesia, and BAL fluid (BALF) collected by rinsing the right middle lobe with three aliquots of normal saline. The second and third aliquots were pooled and used for research purposes, as they provided the most reliable yield in volume and BALF cells (2). Samples were placed on ice immediately after collection. BALF culture was performed as part of routine clinical care in accordance with local guidelines, and results extracted from subjects’ patient records. Remaining BALF was homogenized with a 19G needle, and centrifuged 10 minutes at 800xg to pellet cells. Cells were washed with PBS containing 2.5 mM EDTA, centrifuged, and counted. For flow cytometry, 50.000-200.000 BALF cells were used per staining panel, and fixed with LyseFix (BD Biosciences) prior to acquisition. Antibody panels are listed in **Supplementary Table 1**.

### 2.4 Blood sample collection and processing for flow cytometry

Blood was collected in EDTA tubes and kept on ice until centrifugation for 10 minutes at 800xg, after which plasma was removed, and cells were washed once in PBS with 2.5 mM EDTA. Staining was performed on 50 μL blood, after which erythrocytes were lysed and leukocytes were fixed in one step using LyseFix, prior to acquisition by flow cytometry.

### 2.5 Flow cytometry acquisition and analysis

Acquisition was performed with an LSR Fortessa (BD Biosciences). Measures taken to harmonize procedures and allow comparison of measurements over time, include standard operating procedures and calibration of the flow cytometer to target values using beads. Analysis was performed using FlowJo (BD Biosciences). Neutrophils were defined as CD45^+^ CD66b^+^ CD115^-^. Percentage of BALF neutrophils (BALF %Neu) was expressed as the percentage of CD66b^+^ CD115^-^ events out of total CD45^+^ events. AMs were defined as CD45^+^ CD115^+^ CD33^+^. Expression levels of investigational markers (CD16, CD163, CD91, CD36, PD-1, CD47 and SIRPα) were expressed as either the median fluorescent intensity (MFI) of the total AM population, or as the percentage of AMs positive for each marker. A detailed gating strategy is provided in **Supplementary Figures 1, 2**.

### 2.6 Statistical analysis

Statistical analysis was performed using Prism 9 (GraphPad) and R (https://cran.r-project.org/). Multivariate linear regression models were used to assess the effect of several clinical parameters on different AM marker expression levels. Log-transformed MFI data were used as dependent variables, with age, sex, residual CFTR function, lumacaftor/ivacaftor treatment, positive BALF culture and BALF %Neu as independent variables. To correct for multiple comparisons in the regression model, the cut-off for statistical significance was determined at p < 0.0071. To compare expression levels of investigational markers between age groups, Kruskal-Wallis and Mann-Whitney tests were used. Spearman correlation was calculated to determine associations between flow cytometric outcomes and BALF %Neu. Wilcoxon signed-rank test was used to compare paired BALF and blood data.

## 3 Results

### 3.1 Demographic data

**Table 1** summarizes the demographics of the I-BALL cohort. A total of 50 samples from 38 unique subjects were collected. Approximately half of the subjects (48%) were homozygous for the ΔF508 mutation, and 22% of subjects had at least one known residual function mutation. A high proportion of BALF cultures positive for bacterial pathogens (62%) was observed, 6% of cultures were positive for a fungal pathogen. A subset of I-BALL subjects (28%) were receiving lumacaftor/ivacaftor at the time of sample collection, with treatment duration ranging from 7 days to 34.8 months.

**Table 1:** Cohort demographics.

	1 year (n = 16)	3 years (n = 20)	5 years (n = 14)	All (n = 50)
Sex Male (%) Female (%)	7 (44%) 9 (56%)	10 (50%) 10 (50%)	6 (43%) 8 (57%)	23 (46%) 27 (54%)
CFTR mutation (%) Homozygous ΔF508 Heterozygous ΔF508	6 (38%) 5 (31%)	10 (50%) 8 (40%)	8 (47%) 4 (29%)	24 (48%) 17 (34%)
Residual CFTR function (%)	4 (25%)	5 (25%)	2 (14%)	11 (22%)
Lumacaftor/ivacaftor therapy (%) Duration^a^	0 -	8 (40%) 11.7 (5.4 – 16.6)	6 (43%) 20.4 (0.2 – 34.8)	14 (28%) 15.4 (0.2 – 34.8)
BALF culture Bacteria^b^ *P. aeruginosa* Fungal^c^ * Aspergillus* spp.	11 (69%) 0 0 0	11 (55%) 0 1 (5%) 1 (5%)	11 (79%) 0 2 (14%) 1 (7%)	33 (62%) 0 3 (6%) 2 (4%)

a Mean treatment duration (range) in months.

b Cultures were positive for either one or several of the following pathogens: *Haemophilus influenzae* (12), *Haemophilus haemolyticus* (3), *Klebsiella variicola* (1), *Moraxella catarrhalis* (4), *Staphylococcus aureus* (20), *Streptococcus pneumoniae* (2), *Streptococcus pyogenes* (1).

c Cultures were positive for either one or several of the following pathogens: *Aspergillus fumigatus* (2), *Penicillium* spp (2).

### 3.2 Lung disease severity varies widely within same-age subjects

**Figure 1:**
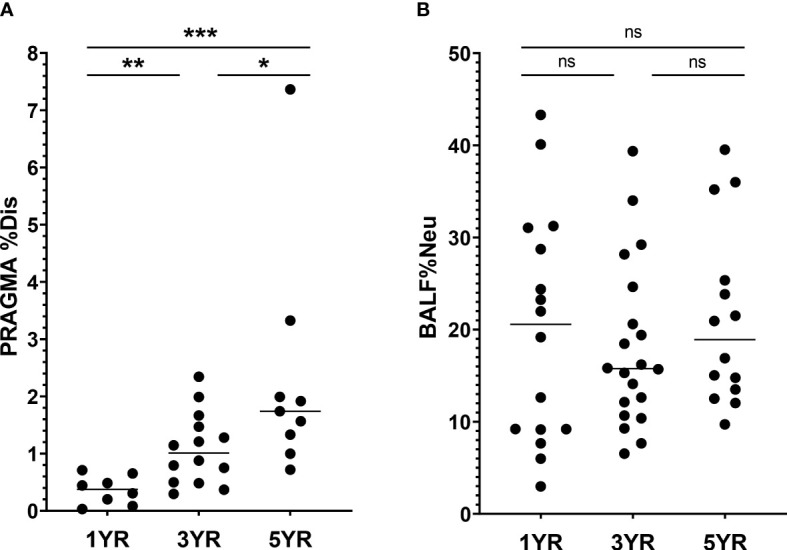
Lung disease parameters in CF patients ages 1, 3 and 5 years in I-BALL cohort **(A)** Structural lung damage across age, measured using the Perth Rotterdam Annotated Grid Morphometric Analysis for CF (PRAGMA-CF) score (n = 31). PRAGMA-CF %Disease (PRAGMA %Dis) is calculated as %bronchiectasis + %mucus plugging + %airway wall thickening and expressed as percentage of total lung volume. **(B)** Frequency of neutrophils in BALF (BALF %Neu) was calculated from flow cytometric data as the percentage CD66b+ neutrophils of total BALF CD45+ leukocytes (gating strategy provided in **Supplementary Figure 1**). Dots represent individual subjects, and the horizontal line in each graph the median. ns, p > 0.05, * p < 0.05, **p < 0.005, ***p < 0.0001.

The PRAGMA-CF %Disease (PRAGMA %Dis) score quantifies structural lung damage in CF subjects, and encompasses both cumulative damage such as bronchiectasis, as well as abnormalities that can be (partially) reversible, such as mucus plugging. In line with earlier reports, PRAGMA %Dis increased with age, with mean scores (± SD) of 0.36 ± 0.2%, 1.08 ± 0.6% and 2.33 ± 1.9% at ages 1 year, 3 years and 5 years respectively (p = 0.0004, **Figure 1A**). Similarly, PRAGMA %Bronchiectasis increased with age, from 0.0% at age 1 to 0.56 ± 1.1% at age 5 (p = 0.01, **Figure S3A**). Severity of airway inflammation at the time of sampling was assessed using the abundance of neutrophils, expressed as a percentage of total BALF leukocytes (BALF %Neu). Overall, mean BALF %Neu was 19.5 ± 10.2% and did not differ between age groups. Rather, we observed a high variability of BALF %Neu in all three age groups (**Figure 1B**). The similar BALF %Neu in all age groups underlines that inflammation is present from an early age. The observed difference in trends between PRAGMA scoring and BALF %Neu further emphasizes the fact that the PRAGMA score is mostly a marker of structural lung damage that has accumulated up to that point in time, while BALF %Neu is a marker of inflammation specific to the time of sampling. Some prior studies reported a correlation between BALF %Neu and PRAGMA %Dis in cross-sectional analysis, while others have not (16, 17). In our cohort, the relationship between BALF %Neu and PRAGMA %Dis showed a positive trend, albeit not statistically significant (**Figure S3B**). Culture positivity increased with age, with the percentage of BALF cultures positive for at least one bacterial or fungal pathogen increasing from 69% at age 1 to 79% at age 5 (p = 0.01, **Table 1**). However, neither BALF %Neu nor PRAGMA %Dis differed between subjects with positive versus negative BALF cultures (**Figure S3C-D**).

Together, these data illustrate that even in the earliest stages of CF airway disease, there is already a great variability between patients with regards to neutrophilic inflammation, infection status and structural lung damage. This indicates that age alone is not the only predictor of CF airway disease progression, and other factors should be taken into account when investigating changes in AM marker expression.

### 3.3 Age and BALF %Neu affect AM expression of CD163, CD91 and SIRPα

We investigated the expression of a variety of scavenging and phagocytosis-related markers on AMs, canvassing scavenger receptors (CD163, CD91, and CD36), opsonin receptor CD16, and immune checkpoint receptors PD-1 and SIRPα and the SIRPα ligand CD47. Expression of investigational markers was assessed both by MFI of the total AM population, as well as the percentage of AMs positive for each respective marker. Expression of AM surface markers can be affected by a range of factors, including genotype, age, sex, environmental exposure, medication, inflammation and infection status. To identify potential factors contributing to changes in AM expression of scavenging and phagocytosis-related markers, we performed a multivariate linear regression model to predict expression of each investigational marker, using age, sex, residual CFTR function, lumacaftor/ivacaftor treatment, culture status and BALF %Neu as independent variables (**Table 2**). This model showed that CD163 and SIRPα expression were affected by age. Additionally, CD163 and CD91 were affected by BALF %Neu. Interestingly, none of the investigated markers were significantly impacted by modulator treatment, residual function or positive cultures.

**Table 2:** Multivariate linear regression model.

	Age	Sex	Residual function^a^	Lu/iva treatment^b^	Culture positive^c^	BALF %Neu^d^
MFI of total airway macrophage population						
CD16	0.5204	0.7658	0.4551	0.3025	0.1136	0.2539
CD163	**0.0015**	0.0879	0.0103	0.6787	0.1785	0.062
CD91	0.2562	0.1645	0.6655	0.2902	0.2481	0.0214
CD36	0.0766	0.5862	0.4237	0.8363	0.4386	0.4453
PD-1	0.1799	0.8991	0.0573	0.9607	0.8245	0.1342
SIRPα	**0.0039**	0.0866	0.3433	0.2143	0.3806	0.4338
CD47	0.4839	0.5722	0.4885	0.3289	0.7867	0.5822
% of airway macrophages positive						
CD16	0.1579	0.8422	0.6491	0.0956	0.7970	0.4223
CD163	0.0530	0.1409	0.2073	0.8207	0.1155	**0.0066**
CD91	0.2858	0.1712	0.8079	0.1467	0.0991	**0.0028**
CD36	0.1074	0.8796	0.5673	0.6033	0.1450	0.1856
PD-1	0.5165	0.1570	0.2565	0.8015	0.5373	0.9644
SIRPα	**0.0023**	0.1458	0.3977	0.8645	0.0350	0.3611
CD47	0.2522	0.7983	0.6523	0.2267	0.3550	0.3623

Numbers depicted are p values, numbers in bold indicate p value below the cut-off for statistical significance, taking into account multiple comparisons correction (p < 0.0071).

a Subject has at least one known residual function mutation.

b Subject was receiving lumacaftor/ivacaftor treatment at time of sample collection.

c BALF culture was positive for either bacterial of fungal pathogen as described in **Table 1**.

d Frequency of neutrophils in BALF (BALF %Neu) was calculated from flow cytometric data as the percentage CD66b^+^ neutrophils of total BALF CD45^+^ leukocytes (gating strategy provided in **Supplementary Figure 1**).

PD-1, programmed cell death protein 1; SIRPα, signal regulatory protein alpha.

### 3.4 Scavenger receptor expression on AMs decreases with age and increased neutrophilic inflammation

**Figure 2:**
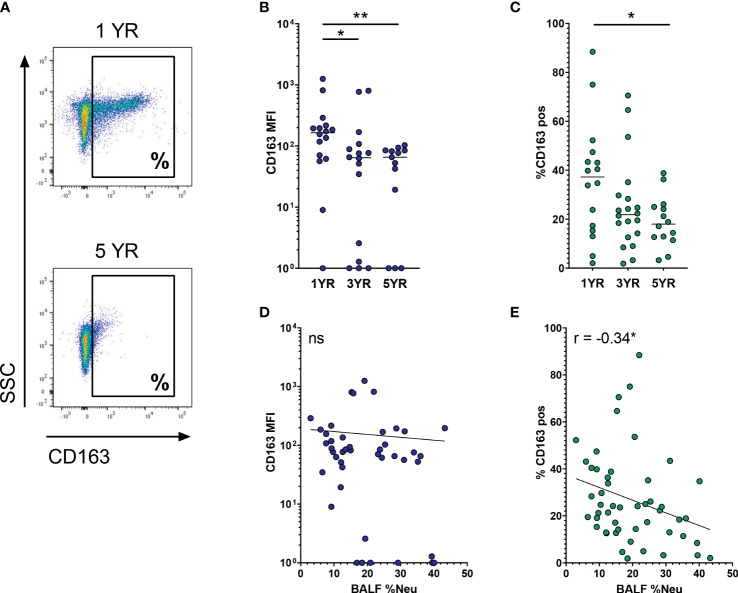
CD163 expression on airway macrophages decreases with age and increased neutrophilic inflammation. Airway macrophages (AMs) were identified as CD45^+^ CD66b^-^ CD115^+^ CD33^+^ cells. Frequency of neutrophils in BALF (BALF %Neu) was calculated from flow cytometric data as the percentage CD66b^+^ neutrophils of total BALF CD45^+^ leukocytes (gating strategy provided in **Supplementary Figure 1**). **(A)** Representative flow cytometry plot depicting CD163 expression on AMs in a 1 year old subject and a 5 year old subject. CD163 expression levels were expressed as either the median fluorescent intensity (MFI) of the total AM population **(B, D)**, or the percentage of AMs positive for CD163 **(C, E)**. **(B, C)** Expression of CD163 on AMs across age groups. Kruskal-Wallis test and Mann-Whitney test were used to compare age groups. Dots represent individual subjects, and the horizontal line in each graph the median (n = 45 for MFI, n = 50 for percentages). **(D-E)** Spearman correlation was used to assess the correlations between BALF %Neu and CD163 expression. Lines depict linear regression. ns, p > 0.05, *p < 0.05, **p < 0.005.

CD163 is a high-affinity SR for pro-inflammatory hemoglobin-haptoglobin complexes, and its expression is associated with the pro-resolution M2 cells. Multivariate analysis indicated that CD163 expression was impacted both by age and BALF %Neu. Indeed, when comparing CD163 expression on AMs between age groups (representative flow plot in **Figure 2A**), a decrease is observed both with respect to MFI of the total AM population (**Figure 2B**), as well as in the percentage of AMs positive for CD163 (**Figure 2C**). The mean percentage of CD163^pos^ AMs drops from 35.9 ± 22.9% at age 1 to 19.1 ± 10.1% at age 5 (p = 0.005), with CD163 expression showing a markedly lower variability in higher age groups. In addition to decreased expression with age, AM CD163 levels were also impacted by BALF %Neu. While increased BALF %Neu does not correlate to changes in MFI of the total AM population (**Figure 2D**), it does show a negative correlation with the percentage of CD163^pos^ AMs (r = -0.34, p = 0.016, **Figure 2E**).

**Figure 3:**
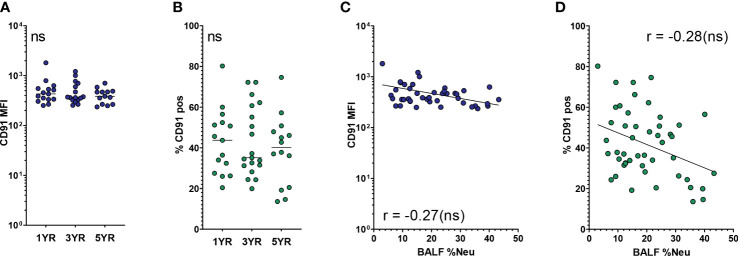
CD91 expression on airway macrophages trends towards decrease with neutrophilic inflammation but not with age. Airway macrophages (AMs) were identified as CD45^+^ CD66b^-^ CD115^+^ CD33^+^ cells. Frequency of neutrophils in BALF (BALF %Neu) was calculated from flow cytometric data as the percentage CD66b^+^ neutrophils of total BALF CD45^+^ leukocytes (gating strategy provided in **Supplementary Figure 1**). CD91 expression levels were expressed as either the median fluorescent intensity (MFI) of the total AM population **(A, C)**, or the percentage of AMs positive for CD91 **(B, D)**. **(A, B)** Expression of CD91 on AMs across age groups. Dots represent individual subjects, and the horizontal line in each graph the median (n = 44 for MFI, n = 48 for percentages). Kruskal-Wallis test and Mann-Whitney test were used to compare age groups. **(C, D)** Spearman correlation was used to assess the correlations between BALF %Neu and CD91 expression. Lines depict linear regression. ns, p > 0.05.

Next, we investigated CD91, the other SR indicated in the multivariate analysis to be affected by neutrophilic inflammation. As a receptor for apoptotic cells, CD91 plays a major role in the ability of AMs to clear apoptotic neutrophils and thereby curb ongoing inflammation. Expression of CD91 on AMs was stable across age groups, both in regard to MFI and percentage of CD91^pos^ AMs (**Figures 3A, B**). With increased BALF %Neu, CD91 expression trended towards lower expression in both MFI (r = -0.27, p = 0.079, **Figure 3C**) and percentage CD91^pos^ AMs (r = -0.28, p= 0.053, **Figure 3D**), although in both cases the correlation did not meet statistical significance.

### 3.5 Expression of SIRPα on AMs decreases with age

**Figure 4:**
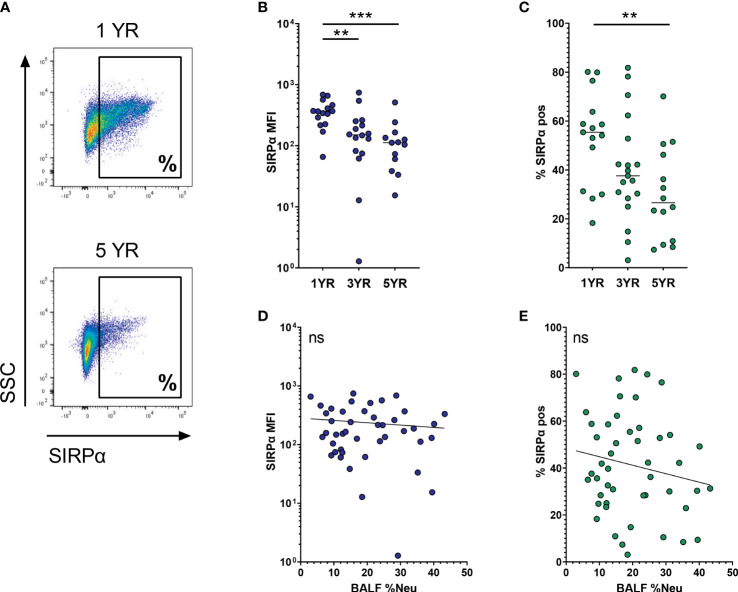
SIRPα expression on airway macrophages decreases with age and but not with neutrophilic inflammation. Airway macrophages (AMs) were identified as CD45^+^ CD66b^-^ CD115^+^ CD33^+^ cells. Frequency of neutrophils in BALF (BALF %Neu) was calculated from flow cytometric data as the percentage CD66b^+^ neutrophils of total BALF CD45^+^ leukocytes (gating strategy provided in **Supplementary Figure 1**). **(A)** Representative flow cytometry plot depicting SIRPα expression on AMs in a 1 year old subject and a 5 year old subject. SIRPα expression levels were expressed as either the median fluorescent intensity (MFI) of the total AM population **(B, D)**, or the percentage of AMs positive for SIRPα **(C, E)**. **(B, C)** Expression of SIRPα on AMs across age groups. Dots represent individual subjects, and the horizontal line in each graph the median (n = 44 for MFI, n = 48 for percentages). Kruskal-Wallis test and Mann-Whitney test were used to compare age groups. **(D, E)** Spearman correlation was used to assess the correlations between BALF %Neu and SIRPα expression. Lines depict linear regression. ns, p > 0.05, **p < 0.005, ***p < 0.0001.

SIRPα is an immune checkpoint receptor that, when bound to its ligand CD47, inhibits AM phagocytic activity. Expression of SIRPα on AMs decreases with age (representative flow plot in **Figure 4A**), and this decrease is most notable when comparing MFI of the total AM population (p = 0.0006, **Figure 4B**). In a similar trend, the percentage of SIRPα^pos^ AMs decreases from 53.0 ± 18.3% at age 1 to 30.2 ± 18.3% at age 5 (p = 0.013, **Figure 4C**). In line with the multivariate analysis, SIRPα expression did not correlate with BALF %Neu, neither when looking at MFI (**Figure 4D**) nor at percentage of SIRPα^pos^ AMs (**Figure 4E**).

### 3.6 Expression of CD36, CD16, PD-1 and CD47 on AMs is not affected by age or neutrophilic inflammation

**Figure 5:**
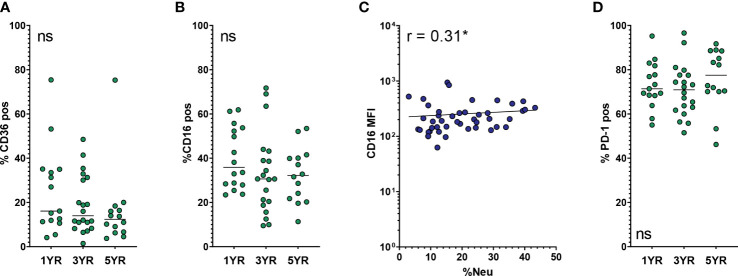
Expression of other phagocytosis-related and immune checkpoint markers on AMs. Frequency of neutrophils in BALF (BALF %Neu) was calculated from flow cytometric data as the percentage CD66b^+^ neutrophils of total BALF CD45^+^ leukocytes (gating strategy provided in **Supplementary Figure 1**). Airway macrophages (AMs) were identified as CD45^+^ CD66b^-^ CD115^+^ CD33^+^ cells. Marker expression levels were expressed as either the median fluorescent intensity (MFI) of the total AM population **(C)**, or the percentage of AMs positive for SIRPα **(A, B, D)**. Dots represent individual subjects, and the horizontal line in each graph the median (n = 44-45 for MFI, n = 49-50 for percentages). **(A)** Expression of CD36 on AMs across age groups. **(B, C)** Expression of CD16 across age groups and BALF %Neu. **(D)** Expression of PD-1 on AMs across age groups. Kruskal-Wallis test and Mann-Whitney test were used to compare age groups. Spearman correlation was used to assess the correlations between BALF %Neu and CD16 expression. Lines depict linear regression. ns, p > 0.05, *p < 0.05.

The third SR included in our analysis is CD36. In addition to its role in lipid scavenging, CD36 is also involved in efferocytosis and recognition of pathogens. AM CD36 expression was not significantly affected by age, BALF %Neu or other covariates in the regression model (**Table 2**). However, there was a low overall expression of CD36 on AMs, with more than 80% of AMs being negative in most subjects (**Figure 5A**).

The OpsR CD16, while not showing any statistically significant association in the multivariate analysis, showed a modest positive correlation with BALF %Neu, but not with age. Percentage of CD16^pos^ AMs was stable across age groups (**Figure 5B**) and with increased BALF %Neu (data not shown), but CD16 MFI of the AM population did increase slightly with BALF %Neu (**Figure 5C**).

The other immune checkpoint receptor included for analysis was PD-1. We previously reported that progressive neutrophilic inflammation in CF airways coincides with an increase of PD-1 expression on AMs (18). High levels of PD-1 might contribute to AM dysfunction seen in CF lungs, as PD-1 negatively affects their phagocytic activity (19, 20). In this cohort however, we found no positive correlation between PD-1 expression and either age or BALF %Neu. Rather, PD-1 MFI was high across age groups and levels of neutrophilic inflammation (data not shown). Of note, the majority of AMs in BALF were PD-1 positive, with a mean of 72.9 ± 11.8% in the entire cohort (**Figure 5D**).

### 3.7 Expression of SRs and phagocytosis-related markers on AMs differs from that on blood monocytes

**Figure 6:**
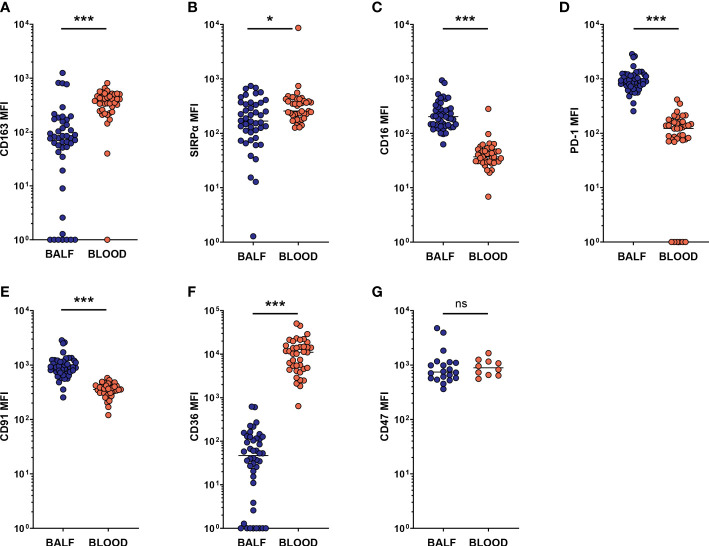
Expression of SRs and phagocytosis-related markers on AMs differs from that on blood monocytes. Airway macrophages (AM) in BALF were identified as CD45^+^ CD66b^-^ CD115^+^ CD33^+^ cells. Blood monocytes (BM) were identified as CD45^+^ FSC^INT^ SSC^INT^ CD66b^-^ CD115^+^ cells (gating strategies provided in **Supplementary Figures 1, 2**). Surface marker expression was expressed as the median fluorescent intensity (MFI) of the total AM or blood monocyte population. Dots represent individual subjects, and the horizontal line in each graph the median. **(A-G)** comparisons of respective marker expressions on AM and BM (n = 44-45 for BALF, n = 39 for blood, for CD47 analysis n = 20 for BALF and n = 10 for blood). Wilcoxon’s test was used for comparison between AM and BM. ns, p > 0.05, *p < 0.005, ***p < 0.0001.

Recently, recruited monocytes and monocyte-derived macrophages were shown to be a major driving force in CF airway inflammation (21). While our study was not set up to differentiate between tissue-resident and recruited macrophage populations, it is reasonable to assume that a subset of the macrophages included in the AM population, as defined by our gating, originates from peripheral blood. To further investigate the difference between macrophages in BALF and their blood counterparts, we next compared the expression of the investigational markers between AMs and peripheral blood monocytes. Compared to blood monocytes, BALF AMs had lower MFIs for CD163 and SIRPα (p < 0.0001 and p = 0.012, respectively, **Figures 6A, B**). On the other hand, expression levels of CD16 and PD-1 were one order of magnitude higher on AMs than on blood monocytes (both p < 0.0001, **Figures 6C, D**), while a less pronounced but nevertheless significantly higher expression of CD91 (p < 0.0001, **Figure 6E**) was also observed on AMs. A striking difference in CD36 expression was observed, with levels on blood monocytes more than two orders of magnitude higher than on AMs (p < 0.0001, **Figure 6F**). Finally, no difference in CD47 expression was observed between BALF AMs and blood monocytes (**Figure 6G**).

## 4 Discussion

In this study performed on fresh BALF macrophages from a pediatric CF cohort, we show that higher age is associated with lower AM expression of SR CD163 and immune checkpoint receptor SIRPα, and that an increase in neutrophilic inflammation in the airways also coincides with a lower expression of CD163. To our knowledge, this is the first report of scavenging and phagocytosis-related marker expression on AMs in the earliest stages of CF airway disease.

The decreased expression of CD163 on AMs, as well as the trend towards decreased CD91 expression with increased BALF %Neu, provides new insight into the possible mechanisms for the reported impaired phagocytic function of CF AMs. An inability to clear the airway lumen of apoptotic neutrophils and other debris is likely to perpetuate pro-inflammatory signaling. Although further functional experiments are needed to confirm a causal relationship, it is likely that the observed changes in SR expression impact scavenging/phagocytic function as shown previously by mechanistic immunology studies. Indeed, blocking of CD163 via an antibody impacts hemoglobin scavenging and pathogen sensing (22, 23), and suppression of CD91 transport to the macrophage cell membrane reduces efferocytic function (24).

In addition to scavenging/phagocytic function, our findings suggest a broader dysfunction of CF AMs, since SR ligand binding can induce either pro-inflammatory or pro-resolution responses, depending on context and co-receptors, which allows AMs to adapt their response to a wide range of stimuli (12, 25). Depending on environmental cues, macrophages can adopt different phenotypes, conventionally divided into M1 (or pro-inflammatory), versus M2 (or pro-resolution). It is precisely that flexibility that allows macrophages to perform their pivotal role in maintaining immune homeostasis. Importantly, such polarization states are not fixed, as macrophages are highly plastic and able to switch from one activation state to another (26–28). CD163 expression is associated with pro-resolution M2 macrophages, and the decrease in CD163 expression we observed is in line with studies reporting an M1-skewed phenotype or impaired M2 skewing in CF AMs (29, 30). In contrast, higher frequencies of CD163^pos^ macrophages are reported in other chronic lung diseases such as asthma and COPD (31, 32). The hemoglobin-haptoglobin uptake that is mediated through CD163 results in the production of several metabolites such as Fe^2+^, CO and biliverdin. Each of these metabolites has been attributed resolution-promoting properties, suggesting that a decreased expression of CD163 could impair the ability of AMs to counter inflammation (33). Indeed, CD163 deficiency has been shown to enhance inflammation in arthritis and LPS-induced shock in mice (34, 35). Airway disease in this early CF cohort is still relatively mild, as demonstrated by low PRAGMA %Dis scores and the absence of *P. aeruginosa*. The relationship between AM SR expression and airway disease progression is a subject of interest for future investigation.

The overall lower expression on AMs than on blood monocytes of CD36 was striking. CD36 is an important SR and fatty acid translocator that is abundant on macrophages in many compartments (36). CD36 expression on steady-state human AMs appears low but is upregulated upon PPARγ activation as occurs during bacterial infection, or in lung diseases such as silicosis and interstitial lung disease (37–41). CD36 is a flexible tool for macrophage function, promoting either pro-inflammatory or pro-resolution activity depending on context and co-receptor stimulation (25, 42). CD36 upregulation by AMs is required for adequate phagocytosis of apoptotic cells (37), and CD36 deficiency leads to impaired M2 skewing in bone marrow-derived macrophages (43). Additionally, CD36 has been shown to play an important role in host protection against infections with *S. pneumoniae* and the key CF pathogen *S. aureus* (39, 44). We speculate that low CD36 expression in CF AMs, or the inability to upregulate CD36, may negatively impact their ability to combat infection, clear apoptotic neutrophils and restore homeostasis.

The negative correlation between age and SIRPα expression suggests that the impaired phagocytic function observed in CF macrophages is likely not due to negative regulation through the CD47-SIRPα axis. A comparison between infant and adult AMs showed equally high levels of SIRPα expression, making it less likely that the decreased SIRPα expression we observe is a physiological change that occurs specifically in infants (45). Indeed, a recent study showed that acute pulmonary inflammation can result in prolonged phagocytic dysfunction in AMs, with low AM SIRPα expression being associated with higher disease severity scores and a higher risk of hospital-acquired pneumonia. This suggests that low SIRPα could be a sign of immunological paralysis of CF AMs (46). The fact that SIRPα negatively correlates with age but not with BALF %Neu, fits the notion that lower SIRPα expression, once acquired through exposure of AMs to an inflammatory environment, can be a sign of prolonged immune paralysis, as opposed to a more short-lived change that mirrors neutrophilic inflammation at the time of sampling.

PD-1 expression is associated with reduced phagocytic activity and more severe illness in pulmonary inflammation (19, 47), and we recently showed that PD-1 blockade improved AM bacterial killing of ingested *P. aeruginosa* (18). While we did not observe the correlation between neutrophil abundance and AM PD-1 expression from our earlier report in a different cohort of children (18), we did observe that overall PD-1 expression was high in BALF AMs, possibly contributing to impaired AM function. With only one study in macaques showing less than 10% PD-1^pos^ AMs in steady-state (19), it is not very likely that PD-1 is constitutively expressed on AMs. However, PD-1 is expressed upon activation on macrophages in other tissues and diseases, in which it is associated with reduced phagocytic capacity (14, 20, 47). Recently, we showed that PD-1 expression on CF monocytes is not directly impacted by CFTR function, but instead induced by the CF airway microenvironment (18). We therefore speculate that the high frequency of PD-1^pos^ AMs in our study is the result of CF airway inflammation, and that PD-1 expression contributes to AM dysfunction in CF.

Ideally, the expression of the phagocytosis-related markers we investigated would be compared to their expression levels on AMs in age-matched non-CF controls, but acquiring BALF AMs from healthy children aged 1-5 years is near impossible due to the invasive nature of BALF collection. One alternative would be to compare children with CF with children that undergo bronchoscopy for other indications. However, because most indications for bronchoscopy, such as anatomical abnormalities or recurring infections, could also impact the inflammatory environment in the lung, the interpretation of this comparison could prove challenging.

Our findings comparing BALF AMs and blood monocytes suggest that the changes in marker expression we observed are specific to the former. For instance, the low expression of CD36 on AMs is not reflected in their blood counterparts, supporting the hypothesis that AM dysfunction in CF is at least in part locally induced by the microenvironment, as opposed to being inherent to CFTR dysfunction (4). One possible contributor to these phenotypic changes in AMs might be the activity of cleaving enzymes, such as neutrophil elastase (NE). Co-culture of monocyte-derived macrophages or AM with NE results in altered receptor expression, shedding of macrophage surface proteins, as well as impaired phagocytosis and efferocytosis (10, 48, 49). Although we did not control for NE activity in our study, some of the investigated markers have been described to be sensitive to NE cleaving. *In vitro* incubation with human recombinant NE was shown to decrease CD91 and CD163 expression in macrophage cell lines (48, 50). However, the impact of the proteolytic CF airway environment on AM marker expression *in vivo* needs further investigation. Protease inhibitors do not fully mitigate the effect of CF airway fluid on AM marker expression (10). Also, decreased expression of other SRs on CF sputum macrophages was shown to occur at both the protein and the mRNA level, suggesting that not all changes observed relate to enzymatic cleavage at the exofacial surface (13). Additionally, pathogen-derived proteases can promote efferocytosis of apoptotic neutrophils by modulating both the ‘eat me’ and ‘don’t eat me’ signals on neutrophils (51), further illustrating that the effective clearance of neutrophils by AM is subject to a complex interplay of factors. Interestingly, none of the phagocytosis and scavenging related markers we investigated were impacted by positive BALF culture (**Table 2**).

A number of subjects in this study received lumacaftor/ivacaftor treatment at the time of sampling. Mean BALF %Neu was higher in lumacaftor/ivacaftor treated subjects compared to untreated subjects (22.7 ± 7.6 and 17.0 ± 9.3 respectively, **Table S2**). No difference in PRAGMA %Dis was observed between treated and untreated subjects (1.2 ± 0.6 and 1.7 ± 1.6 respectively, **Table S2**), although the short follow-up time after start of therapy necessitates caution in drawing conclusions about progression of structural lung disease. There was a large degree of overlap between both groups for both BALF %Neu and PRAGMA %Dis (**Figure S4**). Together, these findings are in line with reports that pulmonary inflammation still occurs despite lumacaftor/ivacaftor treatment (52, 53). Additionally, lumacaftor/ivacaftor treatment was included as a covariate in multivariate analysis and did not show an effect on marker expression (**Table 2**), indicating that the alterations in AM marker expression observed in early CF airway disease are not significantly ameliorated by lumacaftor/ivacaftor treatment. However, this study was not designed to specifically investigate lumacaftor/ivacaftor treatment and we are likely underpowered to make definitive conclusions. The observation that airway inflammation in very young patients is already associated with changes in AM phenotype, serves to support an argument for the early introduction of adequate therapy. In addition to lowering overall airway inflammation through restoring CFTR function in airway epithelium and hereby preventing the effects of prolonged inflammation on AM function, CFTR modulators may also directly improve AM phagocytic function, as has been observed *in vitro* (53). Adequate intervention with CFTR modulators might therefore directly improve AM ability to maintain homeostasis. The effect of the newer elexacaftor/tezacaftor/ivacaftor therapy on airway inflammation and AM marker expression remains to be seen. Therapies other than CFTR modulators, for instance targeted at restoring macrophage functional capacity, may also be of interest.

Taken together, our findings are consistent with the notion that AMs, during the establishment of neutrophilic inflammation in the earliest stages of CF airway disease, gradually become less poised to counter this process. To our knowledge, this is the first description of key scavenging/phagocytic markers on BALF AMs of young children with CF, and of the changes in expression observed as the airway lumen becomes increasingly neutrophil-dominated. Our findings may explain the suggested defects in scavenging ability resulting in perpetuated inflammation, although further research is needed to assess possible causal relationships between our findings and the reduced phagocytic and efferocytic function of AMs in the CF lung. Further studies are also needed to investigate whether these changes are specific to certain AM subsets. Finally, our findings support the hypothesis that AM dysfunction is at least in part induced locally in CF airways, and that lumacaftor/ivacaftor treatment may neither sufficiently attenuate neutrophilic inflammation nor fully restore AM function in patients with CF.

## Data availability statement

The raw data supporting the conclusions of this article will be made available by the authors, without undue reservation.

## Ethics statement

The studies involving human participants were reviewed and approved by Medical Ethics Committee Erasmus MC, Erasmus MC University Medical Centre, Rotterdam, Netherlands. Written informed consent to participate in this study was provided by the participants’ legal guardian/next of kin.

## Author contributions

HJ, RT and WU conceived the project. LS, HH, BM and SE collected patient samples. LS, VG, LP and CS analysed data. LS, VG, CS, LG, RT, LP, HJ and WU interpreted data and drafted the manuscript. All authors contributed to the article and approved the submitted version.
