# Supplementary material for: Airway macrophages display decreased expression of receptors mediating and regulating scavenging in early cystic fibrosis lung disease
Source: Front Immunol. 2023 Jun 29;14:1202009. doi: 10.3389/fimmu.2023.1202009 (PMC10338875; doi:10.3389/fimmu.2023.1202009)
Supplement: Supplementary file 1 [file Presentation_1.pdf]

Supplementary table 1: Antibodies and reagents

	Clone	Company	Catalogue number
Pacific Blue-conjugated anti-human CD41a	HIP8	Biolegend	303714
Pacific Blue-conjugated anti-human CD3	HIT3a	Biolegend	300330
CellTrace Calcein Violet	-	Invitrogen	C34858
BV605 conjugated anti-human CD45	HI30	Biolegend	304042
BV650-conjugated anti-human CD63	H5C6	Biolegend	353026
PerCP-Cy5.5-conjugated anti-human CD16	3G8	Biolegend	302028
PE-conjugated anti-human CD66b	G10F5	Biolegend	305106
PE-Cy7-conjugated anti-human CD115	9-4D2-1E4	Biolegend	347308
APC-Cy7-conjugated anti-human CD33	P67.6	Biolegend	366614
APC-conjugated anti-human CD163	GHI/61	Biolegend	333610
FITC-conjugated anti-human CD91	A2MR-α2	BD Pharmingen	550496
APC-conjugated anti-human CD172a (SIRPα)	15-414	Biolegend	372106
FITC-conjugated anti-human CD279 (PD-1)	EH12-2H7	Biolegend	329904
APC-conjugated anti-human CD36	5-271	Biolegend	336208
APC-conjugated anti-human CD47	CC2C6	Biolegend	323124
Lyse/Fix Buffer	-	BD Biosciences	558049
UltraComp eBeads Compensation Beads	-	Invitrogen	01-2222-42
Rainbow calibration particles, 6 peaks (3.0-3.4µm)	-	Biolegend	422901

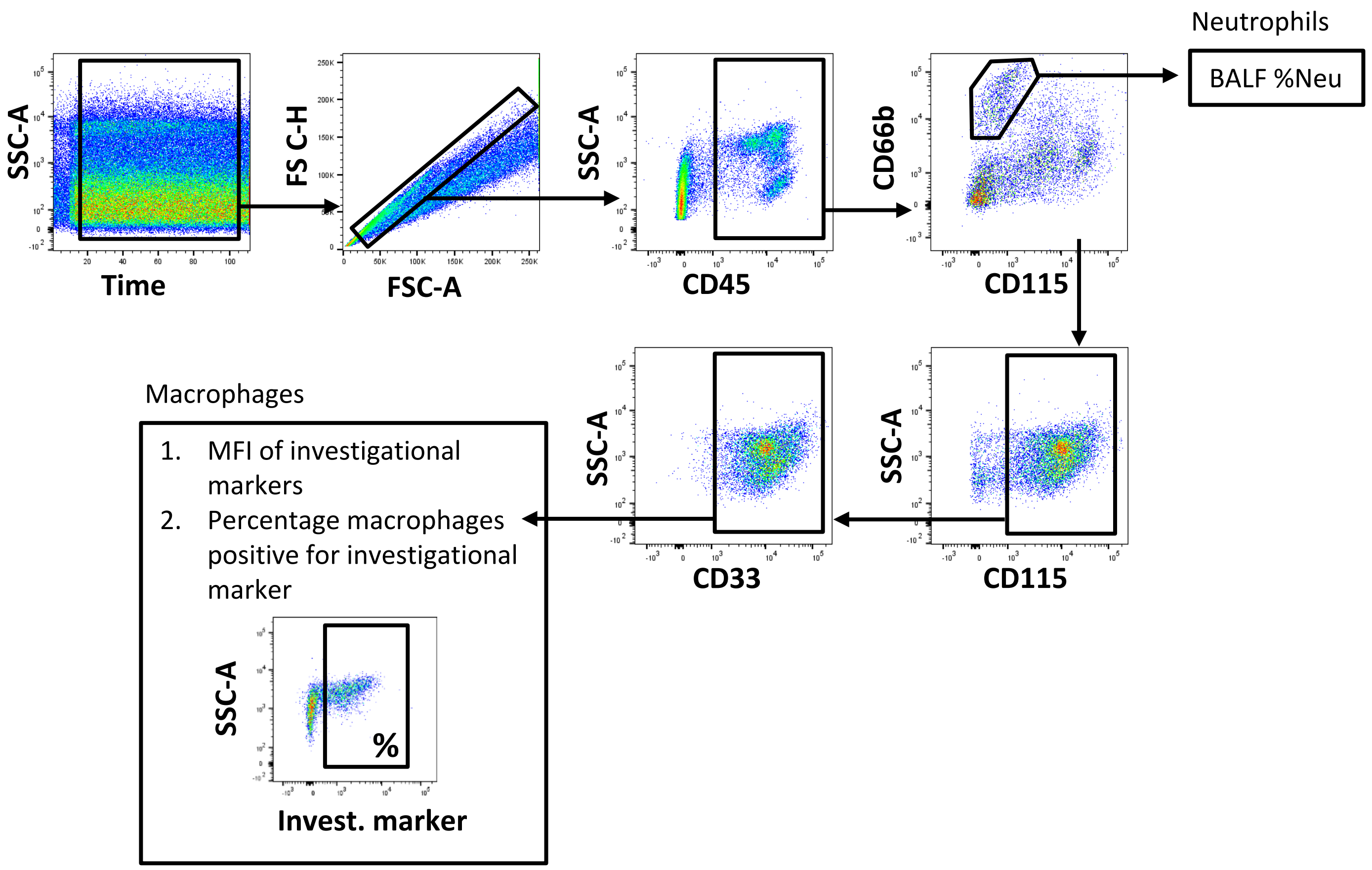

Supplementary figure 1: Gating strategy for bronchoalveolar lavage fluid (BALF) cells.
Flow cytometry was performed on a BD LSR Fortessa, using Rainbow Calibration Particles (BioLegend) to standardize acquisition. Compensation and gating were performed using FlowJo software (LLC, Oregon). First gates exluded flow artefacts and doublet events. BALF leukocytes were identified as CD45+ events. Neutrophils were identified as CD45+ CD66b+ CD115-, and expressed as percentage of total BALF leukocytes. BALF macrophages were identified as CD45+ CD115+ CD33+. Expression of investigational markers (CD16, CD163, CD91, CD36, PD-1, CD47 and SIRPα) was expressed as either the median fluorescent intensity of the total BALF macrophage population, or the percentage of macrophages positive for each respective marker.

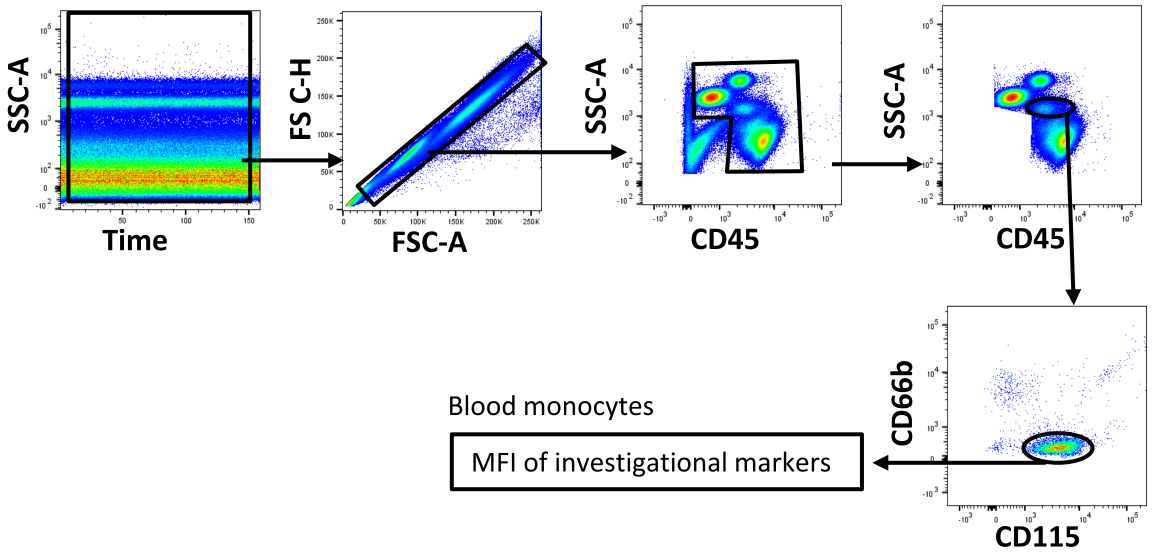

Supplementary figure 2: Gating strategy for peripheral blood monocytes.

Flow cytometry was performed on a BD LSR Fortessa, using Rainbow Calibration Particles (BioLegend) to standardize acquisition. Compensation and gating were performed using FlowJo software (LLC, Oregon). First gates exluded flow artefacts and doublet events. Peripheral blood monocytes were identified as CD45+, CD115+ CD66b- events. Expression of investigational markers (CD16, CD163, CD91, CD36, PD-1, CD47 and SIRPα) was expressed as median fluorescent intensity of the total monocyte population.

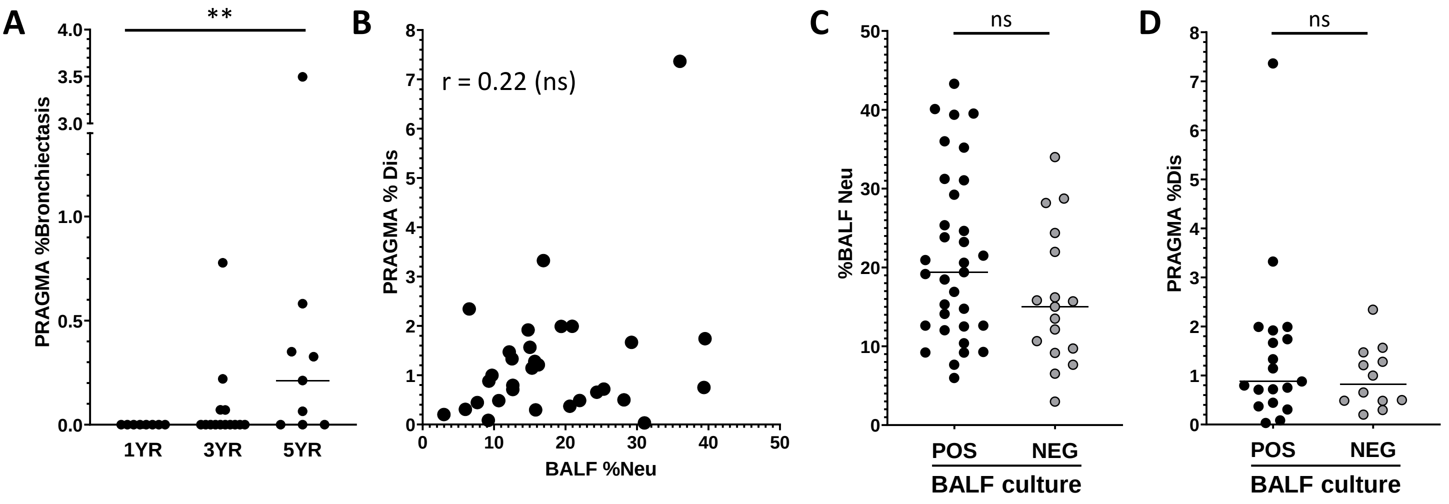

Supplementary figure 3: Additional lung disease parameters and comparisons for culture status.

(A) Percentage of lung volume affected by bronchieactasis (PRAGMA %BE) across age groups, measured using the Perth Rotterdam Annotated Grid Morphometric Analysis for CF (PRAGMA-CF) score and expressed as percentage of total lung volume. (B) Correlation between BALF %Neu and %PRAGMA %Dis was assessed with Spearman correlation. PRAGMA-CF %Disease (PRAGMA %Dis) is calculated as %bronchiectasis + %mucus plugging + %airway wall thickening. (C-D) Comparisons of BALF %Neu and PRAGMA %Dis between culture positive and culture negative subjects. Frequency of neutrophils in BALF (BALF %Neu) was calculated from flow cytometric data as the percentage CD66b^{+} neutrophils of total BALF CD45^{+} leukocytes (gating strategy provided in Supplementary Figure 1). Culture was defined as positive when at least one bacterial or fungal pathogen was found in BALF culture. (ns: $p > 0.05$, ** $p<0.005$).

Supplementary table 2: Comparison of lung disease parameters and culture status in subjects aged 3 and 5 with or without lumacaftor/ivacaftor treatment at time of BALF collection

	Untreated (n=20)	Lumacaftor/ivacaftor-treated (n = 14)	p value[a]
Age 3<br>Age 5	12<br>8	8<br>6	0.99
Sex			
Male	11	5	0.31
Female	9	9	
CFTR mutation			
Homozygous ΔF508	4	14	<0.0001
Heterozygous ΔF508	12	0	
BALF culture positive[b]	9	13	0.009
BALF %Neu[c] (Mean ± SD)	17.0 ± 9.3	22.7 ± 7.6	0.018
PRAGMA %Dis[d] (Mean±SD)	1.7 ± 1.6	1.2 ± 0.6	0.72

[a] Fisher's exact test (age, sex, culture result) or Chi-squared test (mutation type) were used to compare categorical data between groups. Mann-Whitney test was used to compare continuous data (BALF %Neu and PRAGMA %Dis).

[b] BALF culture positive for any bacterial or fungal pathogen.

[c] Frequency of neutrophils in BALF (BALF %Neu) was calculated from flow cytometric data as the percentage $CD66b^+$ neutrophils of total BALF $CD45^+$ leukocytes (gating strategy provided in Supplementary Figure 1).

[d] PRAGMA-CF %Disease is calculated as %bronchiectasis + %mucus plugging + %airway wall thickening and expressed as percentage of total lung volume (n = 16 in untreated group, n = 7 in treated group).

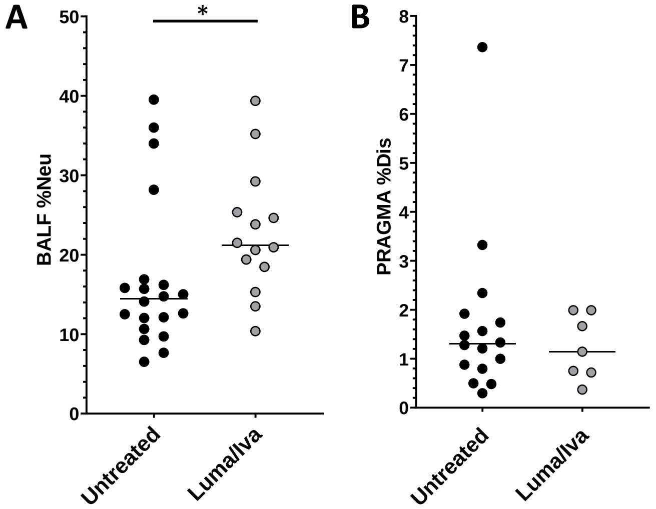

Supplementary figure 4: Comparisons of BALF %Neu and PRAGMA %Dis in subjects age 3 and 5 with or without lumacaftor/ivacaftor treatment

Subjects in the lumacaftor/ivacaftor treated group were receiving modulator therapy at the time of BALF collection, with treatment duration ranging from 7 days to 34.8 months. (A) Frequency of neutrophils in BALF (BALF %Neu) was calculated from flow cytometric data as the percentage CD66b+ neutrophils of total BALF CD45+ leukocytes (gating strategy provided in Supplementary Figure 1). (B) PRAGMA-CF %Disease (PRAGMA %Dis) is calculated as %bronchiectasis + %mucus plugging + %airway wall thickening. Dots represent individual subjects, and the horizontal line in each graph the median. Mann-Whitney test was used for comparison between groups (* $p < 0.05$).
